# Tension-Type Headache: Toward an Integrative Multidimensional Framework for Clinical Stratification and Personalized Management

**DOI:** 10.3390/jcm15082984

**Published:** 2026-04-14

**Authors:** Ana Bravo-Vazquez, Ernesto Anarte-Lazo, Alba Perez-Alvarez, Cleofas Rodriguez-Blanco, Carlos Bernal-Utrera

**Affiliations:** 1 Doctoral Program in Health Sciences, University of Seville, 41009 Seville, Spain; ana.bravo@fisiosurid.es; 2 Physiotherapy Department, Faculty of Nursing, Physiotherapy and Podiatry, University of Seville, 41009 Seville, Spain; alba.perez@fisiosurid.es; 3 Fisiosur I+D, Research Institute, 04630 Garrucha, Spain; ernesto.anarte@universidadunie.com; 4 Faculty of Health, UNIE University, 28015 Madrid, Spain

**Keywords:** tension-type headache, central sensitization, musculoskeletal factors, psychosocial factors, lifestyle, personalized management, clinical heterogeneity

## Abstract

Tension-type headache (TTH) is the most prevalent primary headache disorder worldwide, contributing substantially to individual disability and global socioeconomic burden. Despite its high prevalence, TTH remains clinically heterogeneous, with episodic and chronic forms influenced by the dynamic interplay of peripheral, central, psychosocial, and lifestyle-related mechanisms. Peripheral musculoskeletal factors, including craniocervical muscle alterations and myofascial trigger points, interact with central sensitization processes, while psychosocial stressors, coping strategies, and lifestyle habits such as sleep and physical activity modulate pain perception and chronification risk. Current approaches often address these domains in isolation, limiting therapeutic effectiveness and the understanding of interindividual variability. This narrative review critically synthesizes evidence on the multifactorial determinants of TTH, providing an integrative conceptual framework. We systematically searched PubMed, Scopus, and Web of Science for articles published between 2010 and 2025, including conceptually or methodologically foundational studies outside this range. Relevant studies were selected based on predefined inclusion criteria and synthesized narratively to highlight key mechanisms and contributing factors. The proposed model emphasizes multidimensional assessment, incorporating peripheral musculoskeletal evaluation, central pain modulation, psychosocial profiling, and lifestyle factors, thereby providing a conceptual basis for future personalized management approaches. Recognizing TTH as a dynamic, multidimensional condition may inform clinical assessment and patient-centered interventions, while also highlighting key gaps for future longitudinal and multimodal research aimed at validating the framework and improving individualized therapeutic strategies. The evidence presented is primarily narrative and observational, and clinical applicability should be confirmed in future studies.

## 1. Introduction

Headache is one of the most prevalent and disabling neurological disorders worldwide. In 2021, approximately 2.81 billion people were estimated to be living with some type of headache, with a higher burden among women aged 15 to 49 years, reflecting a particularly significant impact on the working-age population [1]. Due to their high prevalence, disabling potential, and the substantial direct and indirect costs involved, headache disorders are currently considered a global public health priority [2,3].

The diagnostic classification of headaches is established according to the International Classification of Headache Disorders, 3rd edition (ICHD-3), which distinguishes between primary and secondary headaches. Primary headaches are independent clinical disorders without an identifiable underlying structural or systemic cause, whereas secondary headaches are attributed to another medical condition. The primary headache group includes migraine, tension-type headache (TTH), trigeminal autonomic cephalalgias, and other less common disorders [4].

TTH is recognized as the most prevalent form of primary headache, with an estimated global prevalence ranging from 26% to 38%. It is further estimated that between 60% and 80% of the population will experience at least one episode of TTH during their lifetime, while approximately 1–3% will develop chronic forms [5]. Clinically, TTH is characterized by bilateral, pressing or tightening pain of non-pulsating quality and mild to moderate intensity, typically located in the frontal, temporal, or occipital regions, without significant aggravation by routine physical activity and generally without prominent associated symptoms [6].

According to analyses from the Global Burden of Disease study, TTH ranks among the leading causes of years lived with disability worldwide and represents the leading non-fatal cause of neurological disability attributable to pain-related disorders [2]. Although isolated episodes are usually associated with mild to moderate disability, the extremely high population prevalence and recurrence rate make TTH a condition with considerable clinical and socioeconomic impact [6].

Chronic forms, defined by the ICHD-3 as headache occurring on ≥15 days per month for more than three months, account for a disproportionate burden in terms of functional impairment, reduced quality of life, and healthcare utilization, demonstrating an impact comparable to that of other persistent pain syndromes [4,7].

The pathophysiology of TTH involves the interaction of peripheral, central, and psychosocial modulatory mechanisms [8]. Evidence suggests that cranio-cervical muscles—including the upper trapezius, suboccipital, and temporalis muscles—harbor myofascial trigger points (MTrPs) capable of generating referred pain and triggering episodic TTH episodes [9]. Activation of these MTrPs is associated with peripheral nociceptor hyperexcitability, increasing pain perception in response to mechanical stimuli and contributing to initial sensitization [10].

Electromyographic studies and clinical assessments show that baseline activity of the affected muscles is often elevated even at rest, indicating a state of chronic peripheral hypersensitivity that may favor recurrence of painful episodes [11].

In frequent and chronic forms of TTH, persistent pain cannot be explained solely by peripheral factors. Central sensitization represents a key mechanism, characterized by increased neuronal excitability within the central nervous system and reduced descending pain inhibition. Repeated exposure to peripheral muscular pain activates trigemino-cervical circuits and alters central nociceptive processing, creating a cycle of persistent pain [11,12,13].

A central feature of TTH is its marked clinical heterogeneity. Substantial variations exist in the frequency, intensity, and duration of episodes, as well as in the presence of central sensitization, musculoskeletal dysfunction, psychosocial factors, comorbidities, and treatment response [11]. This diversity suggests that TTH should not be interpreted as a homogeneous entity but rather as a complex clinical spectrum resulting from the dynamic interaction between peripheral mechanisms, central neurophysiological processes, and contextual and behavioral modulators. The absence of integrative conceptual models may contribute to fragmented therapeutic approaches and inconsistent clinical outcomes, limiting the effectiveness of interventions targeting isolated components of the problem [12].

Despite the growing body of research on musculoskeletal mechanisms, alterations in pain processing, psychosocial factors, and lifestyle-related variables, the current literature remains partially compartmentalized [13]. A critical integration of these domains is essential to advance toward a multidimensional explanatory framework capable of clarifying the transition from episodic to chronic forms, identifying clinically relevant subgroups, and guiding personalized management strategies.

In this context, the aim of the present study is to conduct a structured narrative and critical review of the available scientific literature on musculoskeletal, psychosocial, and lifestyle-related factors associated with TTH. Epidemiological, clinical, and experimental studies published in scientific databases were examined, prioritizing relevant evidence.

The primary objective is to synthesize current evidence on the multidimensional mechanisms underlying TTH, with a particular focus on explaining its clinical heterogeneity and transition to chronicity. Although variability in treatment response is discussed, this is addressed from a conceptual perspective. The development of individualized interventions is presented as a potential implication of the proposed framework. This work proposes a conceptual model intended to inform future research and support hypothesis generation.

## 2. Materials and Methods

For the development of this structured narrative review, a literature search was performed in PubMed, Scopus, and Web of Science databases to ensure a comprehensive and transparent overview of the available evidence. This approach was intended to support a narrative synthesis, and no formal systematic review methodology or protocol was applied. The most recent search update was performed in February 2026. Only articles published in English and Spanish were considered.

The search strategy combined controlled vocabulary (MeSH terms in PubMed) and free-text terms related to TTH, musculoskeletal factors, psychosocial factors, central sensitization, lifestyle, physical therapy, and pain modulation. Search strings were adapted for each database, combining terms using Boolean operators (AND/OR) in both broad and specific configurations to ensure adequate sensitivity and to capture studies addressing individual domains. The search period was limited to 2010–2025 in order to capture contemporary evidence; however, earlier studies were included when they were considered foundational based on their relevance for defining key concepts, diagnostic frameworks, or underlying mechanisms of TTH. The complete electronic search strategies for all databases are provided in Supplementary Material S1.

The inclusion criteria were: (1) studies conducted in adult populations diagnosed with TTH according to ICHD-2 or ICHD-3 criteria; (2) investigations analyzing factors associated with TTH frequency, intensity, chronicity, or related disability; and (3) studies evaluating musculoskeletal alterations, psychosocial factors, central sensitization, or relevant lifestyle variables. Studies focusing exclusively on migraine or secondary headaches were excluded, as well as studies with major methodological limitations including unclear diagnostic criteria, insufficient sample characterization, or absence of relevant outcome measures related to TTH, which were assessed according to these predefined criteria.

Two reviewers independently (A.B.-V.; C.B.-U.) screened titles and abstracts for relevance. Full-text articles were subsequently assessed for eligibility according to the predefined inclusion and exclusion criteria. Any discrepancies were resolved through discussion and, when necessary, consultation with a third reviewer (E.A.-L.).

**Figure 1:**
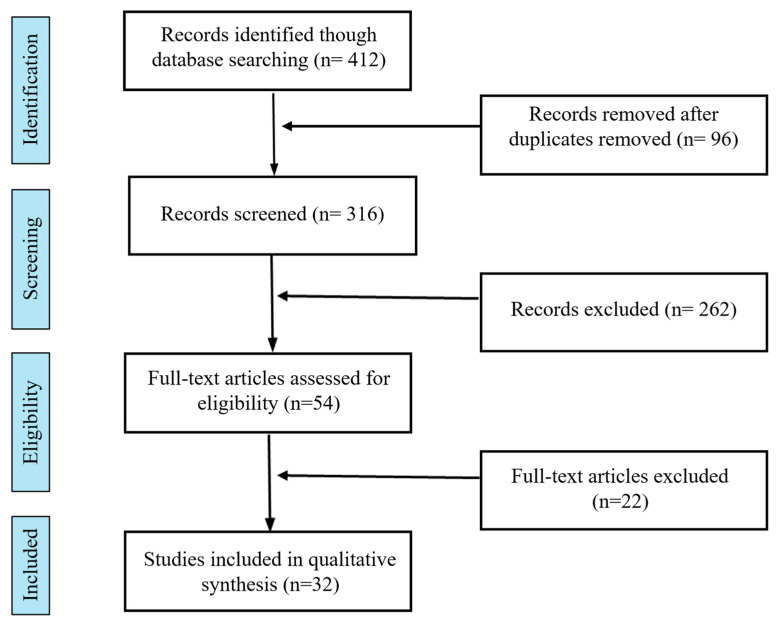
PRISMA-style flow diagram of the study selection process.

A total of 412 records were identified through database searching. After removal of duplicates (n = 96), 316 records were screened by title and abstract, of which 262 were excluded. A total of 54 full-text articles were assessed for eligibility, and 32 studies were finally included in the review. Full-text articles were excluded mainly due to not meeting diagnostic criteria for TTH, lack of relevant outcomes, or insufficient methodological quality. The study selection process is illustrated in a PRISMA-style flow diagram (Figure 1).

Given that the objective of this work is to integrate and contextualize heterogeneous evidence in order to develop a multidimensional conceptual framework, no formal systematic review protocol was followed, and no quantitative meta-analysis was performed. However, efforts were made to ensure transparency and methodological rigor in the search, selection, and synthesis of the evidence. A critical and thematic approach was adopted, aimed at comparing findings, identifying convergences and discrepancies, and highlighting knowledge gaps relevant to clinical practice and future research.

Although a formal risk of bias assessment tool was not systematically applied due to the narrative nature of the review, the methodological quality of the included studies was considered qualitatively, taking into account study design, sample size, and potential sources of bias. Greater weight was given to higher levels of evidence, such as randomized controlled trials, when available. In addition, special attention was given to incorporating recent and diverse literature to ensure an up-to-date and comprehensive synthesis of the topic. Accordingly, findings were interpreted with caution, and causal inferences were avoided unless supported by higher levels of evidence.

Selected studies were organized into thematic categories: (1) musculoskeletal factors; (2) psychosocial factors; (3) central sensitization; and (4) lifestyle habits. This structure enabled an integrated synthesis of the clinical spectrum of TTH and its multidimensional determinants.

## 3. Results

### 3.1. Factors Associated with TTH

Findings are presented as a thematic synthesis. The type and strength of evidence are explicitly indicated where possible, distinguishing between observational studies, experimental research, randomized controlled trials, and meta-analyses to facilitate interpretation of the consistency and robustness of the available evidence.

#### 3.1.1. Musculoskeletal Factors

- Alterations in the Craniocervical Musculature

Observational studies indicate that patients with TTH often present structural and functional impairments in deep cervical and pericranial muscles [14,15,16,17]. Ultrasound and functional evaluations report atrophy and reduced thickness of deep cervical muscles, alongside deficits in endurance, motor control, and fatigue resistance [14,15,16]. Neuroimaging evidence suggests that repeated muscular overload may be associated with higher headache frequency, indicating a potential modulatory role rather than a confirmed causal effect [17].

However, some studies have reported conflicting results, with some cohorts showing no significant differences in muscle thickness or function compared to healthy controls [14,15]. These inconsistencies may be due to small sample sizes, differences in chronicity of TTH, and heterogeneity in assessment methods, emphasizing the need to interpret these findings cautiously.

Electromyography and palpation-based assessments reveal increased resting muscle activity and impaired motor coordination, particularly in chronic TTH [14,15]. While these structural and functional findings are mainly supported by observational evidence, randomized controlled trials (RCTs) and meta-analyses provide stronger evidence for the efficacy of interventions targeting musculoskeletal dysfunction in reducing headache frequency and intensity, without implying that the impairments themselves directly cause TTH [18,19,20,21].

- Myofascial Trigger Points and Referred Pain

Myofascial trigger points (MTPs) are hyperirritable nodules within skeletal muscle that generate local and referred pain [22,23,24]. Case–control studies demonstrate that MTP activation in pericranial and cervical muscles is associated with localized hyperalgesia and increased pain sensitivity [22,25]. A systematic review with meta-analysis confirmed both localized and extracephalic pressure pain hypersensitivity in chronic TTH, with moderate to large effect sizes [25].

MTPs are hypothesized to act as persistent peripheral nociceptive drivers, facilitating central sensitization and sustaining headache recurrence. While this suggests a contributory role, causality cannot be established from observational studies alone [7,13,24]. Interventional RCTs and meta-analyses support the effectiveness of treatments such as manual therapy, dry needling, and myofascial release in reducing headache burden [18,19,20,21].

- Posture and Ergonomics

Poor posture, including prolonged cervical flexion, elevated shoulders, and cervical hyperlordosis, has been associated with TTH in observational studies [15,17,26]. However, some studies did not find strong associations, indicating that postural factors may act as modulators rather than direct causes. Evidence-based guidelines emphasize postural education and cervical strengthening programs as key components of preventive management [27]. Interventions targeting ergonomics, alongside muscle conditioning, may reduce headache frequency according to intervention studies, but causal relationships remain unproven [26,27].

- Critical Perspective

Although musculoskeletal dysfunctions are frequently observed in TTH, the literature suggests they are modulators associated with headache expression rather than isolated causal factors [14,15,16,17,18,19,20,21,22,23,24,25,26,27]. Episodic TTH appears more closely linked to peripheral nociceptive activation of craniocervical muscles, whereas chronic forms involve central sensitization mechanisms and altered pain modulation [8,9,10]. The relative strength of evidence differs across findings: structural and functional impairments are mainly described in observational studies, whereas treatment efficacy is supported by RCTs and meta-analyses. The heterogeneity of study designs, predominance of observational data, and inconsistent findings across cohorts preclude firm causal conclusions. Integrating musculoskeletal findings with psychosocial and lifestyle factors is essential to understand the heterogeneity in clinical presentation and therapeutic response [8,10,13]. Future high-quality RCTs and longitudinal studies are needed to clarify the relative contribution of musculoskeletal factors within this multifactorial framework.

#### 3.1.2. Psychosocial Factors

Psychological stress is conceptualized here as a psychoemotional factor, referring to the individual’s subjective perception of stressors, cognitive appraisal, and emotional responses. Psychosocial factors play a pivotal role in the onset, frequency, and chronification of TTH [7,28,29]. Evidence indicates that the interaction between stress, emotional tension, cognitive appraisal of pain, and occupational conditions influences both pain perception and treatment outcomes. However, most evidence is observational, and findings are not entirely uniform; some studies report weaker or non-significant associations, particularly in smaller cohorts or cross-sectional designs.

- Stress and Emotional Tension

Psychological stress, understood as a subjective psychoemotional experience, is among the most commonly reported triggers for TTH episodes. Longitudinal and cross-sectional studies have demonstrated that sustained exposure to occupational, familial, or social stressors is associated with higher headache frequency and greater pain intensity [30,31,32,33]. Causality cannot be established from these studies alone, and individual coping strategies may modulate this relationship. Mechanistic evidence supports pathways involving prolonged hypothalamic–pituitary–adrenal axis activation and sympathetic nervous system dysregulation, but most data derive from observational studies, limiting causal inference. Evidence from stress-targeted intervention trials (e.g., stress management programs) provides moderate-quality support for improving headache outcomes.

- Anxiety and Depression

Anxiety and depressive disorders are frequently comorbid in chronic TTH [7,34]. Although direct causality remains unproven, psychiatric comorbidity is associated with intensified pain perception, diminishes quality of life, and limits the effectiveness of both pharmacological and non-pharmacological interventions. Most associations come from observational studies, but intervention trials targeting anxiety or depression demonstrate that modifying these factors can have clinically meaningful benefits, indicating stronger evidence for treatment effects rather than causation of TTH onset [35].

- Coping Strategies and Pain-Related Cognitions

Pain perception and management strategies strongly modulate TTH severity and chronicity. Passive coping strategies, such as activity avoidance or overreliance on medication, are associated with increased disability and persistent pain. Conversely, active strategies, including relaxation techniques, pacing, and structured activity planning, are associated with improved functional adaptation. While these relationships are primarily derived from observational data, intervention studies on cognitive-behavioral therapy provide stronger evidence that targeted coping interventions reduce headache burden, but causality in headache genesis remains unconfirmed [35].

- Occupational and Social Factors

Work-related demands and social environment significantly influence TTH expression. High physical and cognitive workloads, low job control, and limited social support are associated with increased headache frequency and chronic neck pain [26,28]. Social isolation, low perceived interpersonal competence, and adverse socioeconomic conditions further amplify pain perception and disability. However, most evidence is observational, and some studies fail to show significant effects after adjustment for confounders, indicating that these factors act as modulators rather than direct causes.

- Critical Perspective

Psychosocial factors act both as triggers and amplifiers of TTH [28,29,30,31,32,33,34,35]. The relationship is bidirectional: chronic headache contributes to psychological distress, while stress, anxiety, depression, and maladaptive coping are associated with headache persistence. The relative strength of evidence varies: associations are mainly observational, whereas intervention studies targeting psychosocial domains (e.g., stress management, coping interventions) provide higher-level evidence for treatment effects. The heterogeneity of findings, predominance of observational designs, and differences in measurement tools limit causal inferences. Higher-level evidence, such as intervention trials targeting psychosocial domains, generally supports clinical benefits, but more robust randomized studies are needed to quantify effect sizes and clarify mechanisms. Integrating psychosocial assessment with musculoskeletal, neurophysiological, and lifestyle considerations is essential to capture the multifactorial nature of TTH and to guide individualized treatment planning [8,10,13].

#### 3.1.3. Central Sensitization and Persistent Pain in TTH

**Figure 2:**
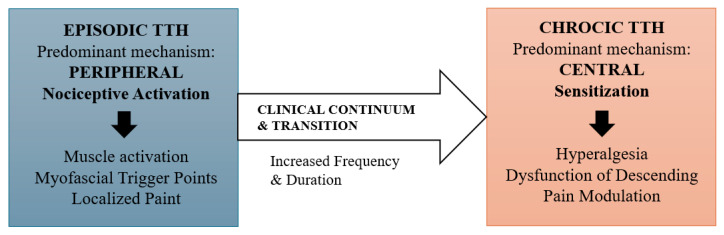
Continuum of tension-type headache (TTH) from episodic to chronic forms.

Central sensitization is considered a key neurophysiological mechanism underlying chronic TTH [25,36]. While peripheral musculoskeletal dysfunction contributes to nociceptive input, repeated and sustained activation of these pathways can induce long-lasting alterations in central pain processing, explaining the persistence and widespread nature of symptoms in chronic forms (Figure 2) [8,9,10].

- Clinical Evidence of Central Sensitization

Meta-analytic evidence demonstrates that patients with TTH exhibit reductions in pressure pain thresholds both in cranial and extracephalic regions, with larger effect sizes in chronic TTH [25]. Functional neuroimaging studies further reveal alterations in pain-processing networks, including heightened activity in the trigeminocervical complex and related cortical regions [37]. These findings indicate associations with central sensitization, but observational and experimental designs preclude definitive causal conclusions.

- Neurophysiological Mechanisms

Central sensitization in TTH involves multiple interrelated mechanisms [35,36]. Synaptic potentiation in the dorsal horn of the spinal cord increases transmission of nociceptive signals from craniocervical musculature, while dysfunction in descending inhibitory pathways may contribute to impaired pain modulation [36,37]. Most mechanistic evidence derives from experimental studies and neuroimaging, while human RCTs examining direct modulation of central sensitization remain limited, indicating a gap in high-level causal evidence.

- Relationship Between Central Sensitization and Peripheral Factors

Central sensitization should not be viewed in isolation [25,36]. Persistent peripheral nociceptive inputs, such as those arising from myofascial trigger points or sustained muscle tension, are associated with facilitation of central amplification. While observational studies support this link, causality cannot be inferred without longitudinal RCTs specifically testing these relationships.

- Interaction with Psychosocial Factors

Psychosocial stressors, including chronic stress, anxiety, depression, and maladaptive pain cognitions, may modulate central sensitization via neuroendocrine and autonomic pathways [35]. These factors may enhance central excitability, reduce descending inhibition, and promote persistence of headache, reinforcing the bidirectional relationship between chronic pain and psychological distress [25,28]. Evidence is mainly observational, and interventional studies targeting stress or coping show only partial improvement in central sensitization markers, indicating modulatory effects rather than confirmed causal pathways.

- Critical Perspective

Evidence supports a continuum model in TTH, in which central sensitization develops progressively in association with repeated peripheral input and psychosocial modulators [8,9,10,25,36]. The relative strength of evidence varies: neurophysiological and imaging data are largely experimental, whereas clinical associations rely mostly on observational studies. Some inconsistencies exist, highlighting the need to interpret findings cautiously and avoid overinterpreting causal links. Much of the mechanistic evidence is derived from experimental pain models and other chronic pain conditions, and its direct applicability to TTH should be interpreted with caution.

#### 3.1.4. Lifestyle Factors and Their Influence on TTH

Lifestyle factors play a significant modulatory role in TTH, influencing frequency, intensity, and chronification. Sleep quality, chronic stress, and physical activity are among the most consistently reported determinants, and they interact dynamically with musculoskeletal, central, and psychosocial mechanisms [38,39,40,41,42,43].

- Sleep and Pain Regulation

Sleep disturbances are associated with higher prevalence, frequency, and intensity of TTH [38,39]. Observational studies indicate that reduced sleep duration and poor sleep quality correlate with increased pain sensitivity and impaired headache control. However, some cohorts failed to find associations, suggesting that sleep may interact with other factors such as stress or comorbidities.

Neurophysiological evidence—largely derived from general pain and sleep research rather than TTH-specific studies—suggests that sleep deprivation disrupts descending inhibitory pain pathways and increases cortical excitability, mechanisms linked to central sensitization [40]. Evidence for intervention (e.g., sleep hygiene programs) comes mainly from small RCTs or quasi-experimental studies, indicating moderate-level support.

- Chronic Stress

Chronic stress is conceptualized in this section as a physiological factor contributing to allostatic load, reflecting the cumulative biological burden of prolonged exposure to stressors. Unlike acute or perceived stress described in the psychosocial domain, chronic stress here refers to sustained neuroendocrine and autonomic dysregulation [42]. Observational studies link prolonged stress exposure to heightened baseline muscle tension, sleep disturbances, and facilitation of central sensitization. RCTs targeting stress management in TTH are limited but suggest improvements in headache frequency, though effect sizes vary. However, a substantial proportion of mechanistic evidence comes from broader stress and pain literature, and direct evidence in TTH populations remains limited.

- Physical Activity and Sedentary Behavior

Regular physical activity is associated with a protective effect against TTH onset and chronification. Population studies demonstrate that low levels of activity are associated with higher headache prevalence, while aerobic and strengthening exercises are associated with a reduced episode frequency and intensity. RCTs examining structured exercise programs provide stronger support for the preventive and therapeutic benefits of physical activity, whereas evidence on sedentary behavior remains largely observational [44,45]. Although these findings are supported by studies in headache populations, part of the mechanistic understanding is extrapolated from general pain research.

- Interaction Between Lifestyle Habits and Pain Mechanisms

Lifestyle factors do not act in isolation but modulate the interplay between peripheral nociception, central sensitization, and psychosocial determinants. Observational studies consistently link combined poor sleep, chronic stress, and inactivity with greater disability, but causal inferences are limited. Interventional evidence from RCTs on sleep, stress, or exercise shows moderate support for improving headache outcomes.

- Critical Perspective

Lifestyle factors should be considered integral modulators rather than isolated triggers of TTH. The relative strength of evidence differs: most associations are based on observational data, whereas targeted lifestyle interventions (sleep programs, stress reduction, structured exercise) are supported by small-to-moderate RCTs, providing higher-level evidence for treatment efficacy. Interventions targeting sleep hygiene, stress reduction, and structured physical activity may mitigate headache frequency, reduce central sensitization, and enhance overall treatment outcomes [38,39,40,41,42,43]. Future high-quality RCTs are needed to confirm causal effects and optimal intervention parameters.

### 3.2. Integrative Conceptual Framework

This narrative review synthesizes the current evidence on the multiple factors associated with TTH, emphasizing the complexity and heterogeneity of this disorder. The findings consistently support the conceptualization of TTH as a multifactorial entity, in which peripheral musculoskeletal, central neurophysiological, psychosocial, and lifestyle mechanisms interact dynamically to shape clinical presentation, disease progression, and therapeutic response [8,10,25,36,38,39,40,41,42,43].

#### Proposal for an Integrative Conceptual Model of TTH

**Figure 3:**
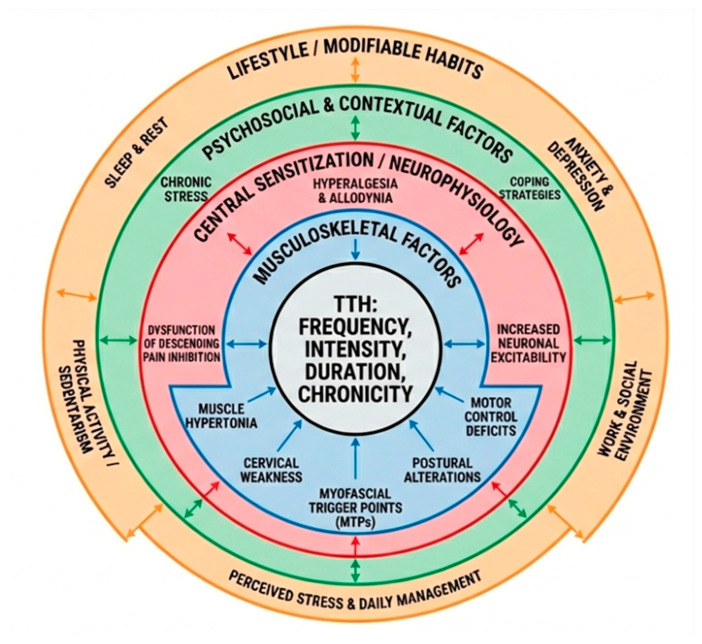
Multidimensional integrative conceptual model of tension-type headache.

Based on the evidence, we propose a multidimensional conceptual model in which TTH emerges from the interplay of four main domains (Figure 3):

- Peripheral musculoskeletal domain: Craniocervical muscles and associated structures capable of generating or amplifying nociceptive input. This includes muscular hyperalgesia, myofascial trigger points, and deficits in strength and motor control [14,15,16,17,18,19,20,21,22,23,24,25,26].
- Central neurophysiological domain: Central sensitization and dysfunction in descending pain modulation, contributing to generalized hyperalgesia and heightened pain responsiveness, particularly in frequent and chronic forms of TTH [36,37].
- Psychosocial domain: Emotional, cognitive, and behavioral factors, including stress, anxiety, depression, and coping strategies, which modulate pain perception and may reinforce chronicity [28,29,30,31,32,33,34,35].
- Behavioral and lifestyle domain: Sleep quality, physical activity, chronic stress exposure, and occupational or ergonomic habits that influence neurobiological regulation of pain and interact with musculoskeletal and central mechanisms [38,39,40,41,42,43].

Within this framework, interindividual clinical variability is explained by the relative contribution and interaction of these domains in each patient. Chronification of TTH is conceptualized not as the result of a single mechanism but as the progressive establishment of maladaptive interactions among peripheral, central, psychosocial, and lifestyle factors. Similarly, differential therapeutic response reflects individual mechanistic profiles, underscoring the need for personalized management strategies.

What is novel about this model is that it explicitly emphasizes the dynamic interactions between all four domains, providing a mechanistic rationale for the observed interindividual variability in TTH presentation, chronification, and therapeutic response. Unlike previous biopsychosocial frameworks, this model integrates measurable clinical indicators for each domain, enabling potential translational application. It highlights that TTH chronification does not result from a single factor but emerges from the cumulative and maladaptive interactions across peripheral, central, psychosocial, and lifestyle domains.

The model also suggests clear pathways for clinical application. Patients may be classified according to dominant mechanistic profiles, such as primarily peripheral musculoskeletal, central sensitization-driven, psychosocially influenced, or mixed presentations. Each domain can be assessed using quantifiable measures, including muscle ultrasound or motor control tests for musculoskeletal function, pressure pain thresholds or quantitative sensory testing for central sensitization, validated stress or anxiety questionnaires for psychosocial factors, and objective monitoring of sleep quality and physical activity for behavioral and lifestyle factors. Understanding a patient’s profile across these domains can inform personalized decision pathways, guiding the prioritization of interventions such as targeted physiotherapy, cognitive behavioral strategies, lifestyle modification, or multimodal approaches.

### 3.3. Clinical Implications

The reviewed evidence suggests that TTH cannot be fully understood through unidimensional models focused exclusively on a single pathophysiological substrate. TTH emerges as a complex and heterogeneous disorder, in which musculoskeletal, psychosocial, neurophysiological, and lifestyle factors interact dynamically, shaping clinical presentation, disease progression, and treatment response [8,10,25,36,38,39,40,41,42,43].

The proposed multidimensional model provides a conceptual perspective that may help inform individualized clinical reasoning, rather than enabling direct clinical stratification. Each patient may present different combinations of musculoskeletal alterations, central sensitization, psychosocial factors, and lifestyle habits, which explains the interindividual variability in the frequency, intensity, and chronicity of TTH episodes.

From a physiotherapy standpoint, clinical assessment is recommended to include specific indicators within each domain, such as cervical muscle strength and motor control, presence of myofascial trigger points and muscular pain, pain thresholds and generalized sensitivity, levels of stress, anxiety, and depression, sleep quality, and physical activity patterns. This approach may help to identify individual profiles that could inform clinical reasoning and hypothesis-driven intervention planning, although its effectiveness has not been directly validated.

Moreover, the model provides a conceptual framework that may support the future development of clinical decision criteria and therapeutic pathways, prioritizing interventions according to the relative contribution of each domain. This integrative perspective also facilitates outcome monitoring, enabling evaluation of changes across multiple domains rather than focusing solely on pain reduction. In this way, the model supports a personalized, multimodal treatment approach, better aligned with the heterogeneous nature of TTH and the variability in clinical response among patients.

In addition to traditional interventions, emerging neuromodulatory approaches targeting the sphenopalatine/pterygopalatine ganglion (SPG/PPG) via transnasal topical delivery have demonstrated anatomical feasibility and preliminary analgesic benefits in headache disorders. While promising, evidence is preliminary, and causal efficacy in TTH remains unproven. Integrating SPG-targeted approaches alongside musculoskeletal, psychosocial, and lifestyle interventions exemplifies a potentially complementary multimodal strategy, based on current associations and emerging evidence. These approaches should be interpreted as preliminary and exploratory within the context of TTH [46].

However, it is important to note that these clinical implications are based on the integration of heterogeneous and predominantly observational evidence. Therefore, they should be interpreted as hypothesis-generating rather than evidence-based recommendations and require validation in future longitudinal and interventional studies.

### 3.4. Limitations

From a critical perspective, this review highlights several limitations in existing literature. There is a predominance of observational studies, with a scarcity of longitudinal designs capable of analyzing clinical trajectories and causal mechanisms. Furthermore, many studies assess factors in isolation without considering their interaction, which restricts a comprehensive understanding of the phenomenon as a whole.

In addition, several limitations related to the review methodology should be acknowledged. First, this study was conducted as a structured narrative review without a formal systematic review protocol, which may limit reproducibility. Although a transparent search and selection process was implemented, the absence of a fully systematic approach increases the risk of selective inclusion bias. Second, no standardized risk of bias assessment tool was applied, consistent with the narrative nature of the review. The methodological quality of the included studies was assessed qualitatively and interpretatively, based on study design and level of evidence, which introduces a degree of subjectivity in the appraisal process and may affect the consistency and robustness of the conclusions. Consequently, the clinical inferences drawn from the evidence should be interpreted with caution. Third, the synthesis was based on a thematic and interpretative approach, without quantitative pooling of results, which may limit the ability to determine the magnitude of effects across studies.

Despite these limitations, synthesizing the available evidence allows progress toward an integrative conceptual model of TTH, in which clinical variability is interpreted as the result of specific combinations of associated factors rather than manifestations of a single homogeneous entity. This approach is particularly relevant for physiotherapy and other clinical disciplines, as it provides a theoretical foundation for individualized therapeutic management and for interpreting variable treatment responses.

From a research standpoint, there is a need for longitudinal and multimodal studies that enable analysis of patients’ clinical evolution, establish causal relationships between domains, and validate the usefulness of the proposed conceptual model in guiding personalized therapeutic strategies.

Overall, this review and the proposed model facilitate understanding of TTH as a dynamic and multidimensional phenomenon, providing a conceptual framework to inform future research and support clinical reasoning.

## 4. Conclusions

The synthesized evidence suggests that TTH cannot be fully understood as a homogeneous entity nor as an exclusively musculoskeletal disorder, but rather as a complex and multidimensional clinical phenomenon involving central neurophysiological processes, psychosocial factors, and lifestyle-related variables. The specific combination and relative contribution of these domains in each individual may contribute to clinical heterogeneity, transition to chronicity, and variability in therapeutic response. These findings should be interpreted within the context of predominantly observational and heterogeneous evidence, and future longitudinal and interventional studies are needed to validate the proposed framework.

## Data Availability

No new data created or analyzed in this study.

## Supplementary Materials

The following supporting information can be downloaded at: https://www.mdpi.com/article/10.3390/jcm15082984/s1, S1: Electronic search strategies.
