# Supplementary material for: Tension-Type Headache: Toward an Integrative Multidimensional Framework for Clinical Stratification and Personalized Management
Source: J Clin Med. 2026 Apr 14;15(8):2984. doi: 10.3390/jcm15082984 (PMC13116980; doi:10.3390/jcm15082984)
Supplement: Supplementary file 1 [file jcm-15-02984-s001.zip › jcm-4238982-supplementary.pdf]

S1. Electronic search strategies

PubMed

The following MeSH terms were applied:

- “Headache, Tension-Type”[MeSH]
- “Musculoskeletal Diseases”[MeSH]
- “Musculoskeletal Pain”[MeSH]
- “Psychological Factors”[MeSH]
- “Stress, Psychological”[MeSH]
- “Central Sensitization”[MeSH]
- “Life Style”[MeSH]
- “Physical Therapy Modalities”[MeSH]
- “Pain Perception”[MeSH]
- “Chronic Pain”[MeSH]

Example search strategy:

("Headache, Tension-Type"[MeSH] OR "tension-type headache")
AND ("Musculoskeletal Diseases"[MeSH] OR "Musculoskeletal Pain"[MeSH] OR "musculoskeletal factors")
AND ("Psychological Factors"[MeSH] OR "Stress, Psychological"[MeSH] OR "psychosocial factors")
AND ("Central Sensitization"[MeSH])
AND ("Life Style"[MeSH] OR lifestyle)
AND ("Physical Therapy Modalities"[MeSH] OR "physical therapy")
AND ("Pain Perception"[MeSH] OR "pain modulation")

Scopus

The search was conducted using free-text terms in the TITLE-ABS-KEY fields.

Example search strategy:

TITLE-ABS-KEY (
"tension-type headache"
AND ("musculoskeletal factors" OR "musculoskeletal pain")
AND ("psychosocial factors" OR "psychological stress")
AND "central sensitization"
AND lifestyle
AND "physical therapy"
AND ("pain modulation" OR "chronic headache")
)

Web of Science

The search was performed using free-text terms within the Topic field (TS), which includes title, abstract, author keywords, and Keywords Plus.

Example search strategy:

```
TS=(
"tension-type headache"
AND ("musculoskeletal factors" OR "musculoskeletal pain")
AND ("psychosocial factors" OR "psychological stress")
AND "central sensitization"
AND lifestyle
AND "physical therapy"
AND ("pain modulation" OR "chronic headache")
)
```
